# Metropolitan Hospital Sunday Fund

**Published:** 1902-08-09

**Authors:** 


### METROPOLITAN HOSPITAL SUNDAY FUND.

Except for representatives of the press, who are generally in full force at this annual gathering, the attendance at the Council Meeting of the Metropolitan Hospital Sunday Fund at the Mansion House on Tuesday, under the presidency of the Lord Mayor, though select and representative, was no larger than might be expected in London during August, many letters of regret for absence having been received.

Minutes of previous meetings having been read by the new Secretary of the Fund (Sir Edmund Hay Currie), the Lord Mayor (Sir Joseph Dimsdale) said it afforded him the greatest pleasure to announce that in that thirtieth year of the Fund's existence the amount realised by the collections had already reached £60,000, and before the end of the year they hoped it would be considerably in excess of any of the twenty-nine preceding years. Mr. George Herring's noble offer to add 25 per cent. to the amount collected had been a great incentive to the congregations in the various places of worship. At St. Paul's, as a result of the special appeal he had made, the sum of £2,129 was collected—ten times more than had been raised before. Prebendary Ridgway's congregation at Christ Church, Lancaster Gate, contributed £1,628; St. Michael's, Chester Square, sent £1,388; and he might mention that since the institution of the Fund Canon Fleming's contributions had totalled over £25,000. Westminster Abbey had yet to come in, but there was no doubt that there was a substantial increase in the amount received from places of worship, not forgetting the help always given by their friends of the Jewish community (a statement received with murmurs of applause) for it is one of the special features of the Sunday Fund that it brings together on the most friendly footing representatives of all shades of religious thought and persuasion.

### A Change in the Basis of Award.

Continuing, Sir Joseph said there were still many hospitals with beds unoccupied—among others he might mention Guy's, and the Metropolitan, Kingsland Road—and it was worthy of consideration whether fresh beds should be opened while existing beds could not be utilised owing to want of funds. They would be asked to sanction the recommendation that the Fund should be expended on the maintenance of patients, and not on bricks and mortar. A great part of the money they received was subscribed by the poorer part of the community, with the idea of providing for the patients and not for the extension of the buildings. They were aware, of course, that 5 per cent. of the amount collected was set apart for surgical appliances, and for these there was a very great demand throughout London. He was glad that in this memorable year, and in his mayoralty, a record collection had been made. He would remind them that during the year they had lost two members of the council—Sir Richmond Cotton by death, and Colonel Francis Hagar by resignation on account of failing health. Both were old and respected members of the Fund, and their loss was much regretted.

Sir Sydney Waterloo moved the adoption of the report of the Committee of Distribution, which was as follows:—

The Committee of Distribution held their first meeting on Tuesday, January 7th, and having at various subsequent meetings examined the reports and balance sheets of the several institutions seeking to participate, made their awards in conformity with Law V.

The committee recommend awards this year to 202 institutions. The amount of collections, including interest from the Court of Chancery, to the present time is £59,600. The distribution of £57,800 to 147 hospitals, etc., and to 55 dispensaries, is now recommended.

This year 204 institutions, or four more than last year, have made applications for grants from the funds at your disposal. The committee of one old-established hospital, not being in immediate want of funds, did not seek to participate. Of this number it was found necessary to invite the attendance of deputations from the governing bodies of 17 institutions. Your committee, after a conference with representatives of 10 hospitals and two dispensaries, where the expenditure appeared to them to be excessive, have reduced their bases, and of five other institutions which did not send deputations to confer with your committee, three had their bases reduced, and in the case of one hospital and one dispensary no award was made. In the case of the two dispensaries the explanations given were satisfactory.

The serious attention of the committee has been drawn to the increasing cost of maintaining the in-patients in several hospitals. This is a matter which they have already considered, and purpose to investigate still further hereafter.

Mr. George Herring having again most liberally offered a donation of £10,000, or to give one-fourth of the amount collected in places of worship, and the latter proposal having been accepted, it is satisfactory to note that a general increase has resulted, and a "record" amount obtained in this memorable year at the places of worship.

The committee have decided to recommend that the "Extraordinary repairs and building improvements" will not in future be included in the bases, so that the money distributed will be wholly awarded to the "maintenance" of the patients.

Five per cent. of the total sum collected is set apart to purchase surgical appliances (in obedience to Law XII.) in monthly proportions during the ensuing year.

In compliance with an order of the Council, and for the special use of its members, Tables of Statistics have been prepared as usual, showing an analysis of the number of beds in hospitals, the cost of patients both at hospitals and dispensaries, the proportionate expense of management, as well as other valuable information, and copies are sent to every member of the Council. (Signed) Joseph C. Dimsdale, Lord Mayor, President and Treasurer; Sydney H. Waterlow, Vice-President; Stamford; William Church; Savile Crossley; James Cundy; Robert Grey; Herman Hoskier; F. H. Norman; Alfred Willett.

The veteran Vice-President and Chairman of the Distribution Committee said he was sure they would sympathise with him in the great pleasure he felt in asking the council to agree with the committee in its report and to sanction the several awards set out in the list in their hands. In a very few weeks the Fund would have reached its thirtieth year, and it was a red-letter day in its history to record the largest subscription they had ever received. In 1870 he had a conversation with the late Canon Miller, who had successfully tried the plan of a Hospital Sunday collection. They called a few friends together, and they met and passed resolutions expressing the feeling that it was desirable to establish a Hospital Sunday in London. In 1873 they had their first collection, which amounted to £27,700. Year by year the amount received had constantly increased, and this year they expected it to reach over £60,000. He thought there could not be better evidence of the confidence of the public in the management of the Fund than the fact that every year they entrusted them with more money to distribute. So far they had received £57,800 as compared with £45,063 last year—an increase of £12,737. The collections in places of worship had all been larger, the increase being relatively largest from the smaller congregations in the suburban districts.

### Increasing Cost of Maintenance.

The labours of the Distribution Committee had that year been unusually heavy. They had received a large number of deputations from various hospitals with a view to making sure of the grounds on which they based their awards. They had been struck by the increase in the cost, in the general hospitals per bed per week, for the maintenance of in-patients. It varied from £1 5s. 6d. to as much as £2 9s. at one of the largest hospitals. The average cost at the general hospitals was £1 14s. 7d., and the committee were convinced—and saw no reason, after their conferences with the various deputations, to vary their opinion—that that was a fair and reasonable amount. They were assured that the Sunday Fund was in no way at variance with, or in competition with, King Edward's Fund—they worked harmoniously together; and the longer he was connected with it the more he felt that the money subscribed to the Sunday Fund was intended for the maintenance of patients, so as to prevent that sad state of things—hospitals with empty beds. They therefore recommended that it should go for maintenance, and not for extraordinary repairs and building. He felt that they were very greatly indebted to the clergy of all denominations for the way they brought the matter before their congregations, and hoped they would all continue to do their best to stimulate collections so as to do better still next year, when they would have a better chance, since there would not be so many other calls.

Lord Stamford seconded. As a junior member of the committee, and therefore largely an onlooker at its work, he could bear witness to the amount of work and the number of attendances involved, and the great care and consideration given to individual cases. One fact that had struck him more than anything else in listening to the discussion with regard to the variation in the cost of beds at different institutions was that high cost by no means meant correspondingly high efficiency.

Sir Henry Burdett supported the resolution, and at the outset wished to point out that at Guy's the unoccupied beds were mainly among those set apart for paying patients. The Metropolitan, Kingsland Road, certainly had a large number of empty beds which might usefully be occupied; and he thought anyone desirous of interesting himself in hospitals might give a lift to the East End by helping that hospital, and so do a real service to the poor. He noticed with approval that the Committee had refused a grant to the Hospital of St. Francis. It was not really a hospital at all, but what was practically a 3d. dispensary. It was a small house which did not fulfil many of the conditions indispensable in a hospital. The whole place was unworthy of the name, and he thought it was a reflection on the health authorities that the name should be applied to it. He thought the Fund should protest against attempts to obtain money for such establishments, and that no institution should be eligible to apply for a grant unless it had been visited by a representative of the Fund. He observed that they had again given a grant to the Jubilee Hospital. He should have voted against that; and as to the Oxygen Home, though he believed it did a useful work in the treatment of ulcers, yet he did not consider it came within the four corners of their scheme. He warmly approved of the important departure involved in the decision not to include expenditure on bricks and mortar in the basis of award. He would suggest that the names of institutions which did not send representatives in response to the invitation to confer with the committee should be given. He was almost inclined to think also that the time had arrived to fix a maximum unit of expenditure per bed. In some institutions expenditure was carried on on such a magnificent scale that it was impossible the public could go on finding money to provide luxuries which were not essential to the proper treatment of disease and which did not hasten recovery. If they helped to check such expenditure they would be doing an immense service not only to the public and the patients, but to the hospitals themselves. One item especially had gone up by leaps and bounds of late years, and that was nursing. At one hospital, a palace—for it was nothing else, and he recognised that it was due to the munificence of a particular donor—had been erected; and the effect on the nurses was as curious as it was remarkable. There was a marble staircase; a dining-room elaborately decorated, and yet the nurses said they were not so comfortable in this glorified building as in much humbler buildings. The nurses declared it was more like a beautiful prison than a home, and its marble magnificence chilled them. They preferred simpler surroundings even if they were a little more crowded together.

The Lord Mayor wished to remove any suggestion of inaccuracy from his remarks about Guy's, and agreed that the empty beds were those reserved for paying patients. But they were beds which, if Guy's had the necessary funds, would be utilised for the benefit of the poor.

Sir Henry Burdett did not wish to be understood as not in favour of paying wards. He only wanted to have the facts correctly. He thought Guy's were probably doing better work with more beds for paying patients than if they were all free.

The motion for the adoption of the report was then put and carried unanimously.

Sir William Church moved a cordial vote of thanks to Mr. George Herring for his generous donation, which had materially assisted in raising the total collections this year to a record amount, and this was seconded by Canon Fleming and carried by acclamation.

The Chief Rabbi (Dr. Adler) proposed, and Mr. Macnamara seconded, the next resolution—a hearty vote of thanks to

Sir Sydney Waterlow and the other members of the Committee of Distribution, for the care bestowed in the preparation of the awards they had recommended. This also was carried with applause.

After a few words of acknowledgment from Sir Sydney, thanks were voted to the press for their advocacy of the Fund, and the Lord Mayor then moved a resolution, not on the agenda, conveying to Sir Edmund Hay Currie, "their new secretary, but very old friend," hearty God-speed in his new office. He was the ideal man for the position, and a veteran in all that pertained to hospital work and endeavour.

Sir Sydney Waterlow seconded this, and it was cordially supported by Sir Henry Burdett, and carried.

Lord Meath then moved a vote of thanks to the Lord Mayor, and in doing so, endorsed, from his experience in Dublin, Sir Henry Burdett's opinion that no hospital should get an award unless it had been favourably reported on after inspection by a member of the Fund. The motion was duly seconded and carried.

### PARTICULARS OF AWARDS.

THE following are the Awards of the Committee of Distribution adopted by the Council of the Metropolitan Hospital Sunday Fund at the meeting on August 5th:—

### 31 GENERAL HOSPITALS.

Charing Cross Hospital, £1,207 10s.; French Hospital, £306 13s. 4d.; German Hospital, £594 3s. 4d.; Great Northern Central Hospital, £1,053 12s. 1d.; Guy's Hospital, £2,875; Hampstead Hospital, £239 11s. 8d.; Italian Hospital, £115; King's College Hospital, £1,437 10s.; London Hospital, £6,181 5s.; London Homœopathic Hospital, £527 1s. 8d.; Phillips' Memorial Homœopathic Hospital, £34 10s.; London Temperance Hospital, £776 5s.; Metropolitan Hospital, £862 10s.; Mildmay Hospital, £287 10s.; Miller Hospital and Royal Kent Dispensary, £287 10s.; North-West London Hospital, £431 5s.; Poplar Hospital, £621 5s. 4d.; Queen's Jubilee Hospital, £95 16s. 8d.; Royal Free Hospital, £1,245 16s. 8d.; St. George's Hospital, £1,916 13s 4d.; S.S. John and Elizabeth Hospital, £143 15s.; St. Mary's Hospital, £2,489 15s. 4d.; St. Thomas's Hospital, £1,339 13s. 4d.; Seamen's Hospital Society, £1,293 15s.; The Middlesex Hospital, £2,309 11s. 8d.; Tottenham Hospital, £347 4s. 2d.; University College Hospital, £1,303 6s. 8d.; Walthamstow, £100 8s. 4d.; West Ham Hospital, £525 17s. 8d.; West London Hospital, £814 11s. 8d.; Westminster Hospital, £1,386 8s. 8d.

### SPECIAL HOSPITALS.

#### 5 CHEST HOSPITALS.

City of London Hospital for Diseases of the Chest, Victoria Park, £958 6s. 8d.; Hospital for Consumption, Brompton, £1,820 16s. 8d.; Mount Vernon Consumption Hospital, Hampstead, £670 16s. 8d.; Royal Hospital for Diseases of the Chest, City Road, £383 6s. 8d.; Royal National Hospital for Consumption, Ventnor, £383 6s. 8d.

#### 17 CHILDREN'S HOSPITALS.

Alexandra Hospital for Hip Disease, W.C., £239 11s. 8d.; Banstead Surgical Home, £23 19s. 2d.; Barnet Home Hospital, £43 2s. 6d.; Belgrave Hospital for Children, S.W., £124 11s. 8d.; Cheyne Hospital for Incurable Children, S.W., £124 11s. 8d.; East London Hospital for Children, Shadwell, E., £766 13s. 4d.; Evelina Hospital for Sick Children, Southwark, S.E., £287 10s.; Home for Incurable Children, Maida Vale, W., £76 13s. 4d.; Home for Sick Children, Sydenham, S.E., £134 3s. 4d.; Hospital for Sick Children, Great Ormond Street, W.C., £1,054 3s. 4d.; North-Eastern Hospital for Children, Hackney Road, N.E., £287 10s.; Paddington Green Hospital for Children, W., £297 1s. 8d.; St. Mary's, Plaistow, E., £249 3s. 4d.; St. Monica's, Brondesbury, N.W., £95 16s. 8d.; Victoria Hospital for Children, King's Road, Chelsea, S.W., £460; Victoria Home, Margate, £47 18s. 4d.; "The Vine," Sevenoaks, £43 2s. 6d.

#### 5 LYING-IN HOSPITALS.

British Lying-in Hospital, Endell Street, W.C., £28 15s.; Clapham Maternity Hospital, £38 6s. 8d.; East End Mothers' Home, £76 13s. 4d.; General Lying-in Hospital, Lambeth, S.E., £57 10s.; Queen Charlotte's Lying-in Hospital, Marylebone Road, W., £479 3s. 4d.

#### 6 HOSPITALS FOR WOMEN.

Chelsea Hospital for Women, S.W., £421 13s. 4d.; Hospital for Women, Soho Square, W., £140 12s; Grosvenor Hospital for Women and Children, Vincent Square, S.W., £172 10s.; New Hospital for Women, Euston Road, W.C., £306 13s. 4d.; Royal Hospital for Children and Women, Lambeth, S.E., £268 6s. 8d.; Samaritan Free Hospital, Marylebone Road, W., £492 6s. 8d.

#### 27 OTHER SPECIAL HOSPITALS.

Cancer Hospital, Brompton, S.W., £524 19s. 8d.; London Fever Hospital, Islington, N., £718 15s.; Gordon Hospital for Fistula, Vauxhall Bridge Road, S.W., £23 19s. 2d.; St. Mark's Hospital for Fistula, City Road, E.C., £143 15s.; National Hospital for the Diseases of the Heart, Soho Square, W., £76 13s. 4d.; Female Lock Hospital, Harrow Road, W.; £306 13s. 4d.; Hospital for Epilepsy, Paralysis, and other Diseases of the Nervous System, Regent's Park, N.W., £47 18s. 4d.; National Hospital for the Paralysed and Epileptic, W.C., £958 6s. 8d.; West End Hospital for Diseases of the Nervous System, £239 11s. 8d.; Central London Ophthalmic Hospital, Gray's Inn Road, W.C., £115; Royal Eye Hospital, St. George's Circus, S.E., £239 11s. 8d.; Royal London Ophthalmic Hospital, City Road, E.C., £862 10s.; Royal Westminster Ophthalmic Hospital, Charing Cross, W.C., £134 3s. 4d.; Western Ophthalmic Hospital, Marylebone Road, W., £47 18s. 4d.; City Orthopædic Hospital, Hatton Garden, E.C., £249 3s. 4d.; National Orthopædic Hospital, Great Portland Street, W., 124 11s. 8d.; Royal Orthopædic Hospital, Oxford Street, W., £134 3s. 4d.; Royal Sea Bathing Hospital, Margate, £287 10s.; St. John's Hospital for Diseases of the Skin, £57 10s.; St. Peter's Hospital for Stone, Covent Garden, £95 16s. 8d.; Central London Throat and Ear Hospital, Gray's Inn Road, W.C., £47 18s. 4d.; Hospital for Diseases of the Throat, Golden Square, W., £249 3s. 4d.; London Throat Hospital, Great Portland Street, W., £28 15s.; Metropolitan Ear, Nose, and Throat Hospital, Grafton Street, W., £28 15s.; Royal Ear Hospital, Frith Street, W., £19 3s. 4d.; Dental Hospital of London, Leicester Square, W.C., £143 15s.; National Dental Hospital, 149 Great Portland Street, W., £38 6s. 8d.

### 28 CONVALESCENT HOSPITALS.

Metropolitan Convalescent Institution, Sackville Street, W., £670 16s. 8d.; Bexhill Convalescent Home, Sackville Street, W., £383 6s. 8d.; All Saints' Convalescent Hospital, Eastbourne, £383 6s. 8d.; Ascot Priory Convalescent Home, £67 1s. 8d.; Charing Cross Hospital Convalescent Home, Limpsfield, £86 5s; Chelsea Hospital for Women Convalescent Home, St. Leonards, £47 18s. 4d.; Deptford Medical Mission Convalescent Home, Bexhill, £28 15s.; Mrs. Gladstone Convalescent Home, Mitcham, £47 18s. 4d.; Friendly Societies' Convalescent Home, Dover, £38 6s. 8d.; Hahnemann Convalescent Home, Bournemouth, £38 6s. 8d.; Hanwell Convalescent Home, £19 3s. 4d.; Herbert Convalescent Home, Bournemouth, £19 3s. 4d.; Herne Bay Baldwin-Brown Convalescent Home, £28 15s.; Homœopathic Convalescent Home, Eastbourne, £19 3s. 4d.; King's College Hospital Convalescent Home, Hemel Hempstead, £172 10s.; Mrs. Kitto's Convalescent Home, Reigate, £67 1s. 8d.; Mary Wardell Convalescent Home for Scarlet Fever, £105 8s. 4d.; Morley House Convalescent Home for Working Men, £210 16s. 8d.; Police Seaside Home, Brighton, £57 10s.; St. Andrew's Convalescent Home, Clewer, £134 3s. 4d.; St. Andrew's Convalescent Home, Folkestone, £239 11s. 8d.; St. John's Home for Convalescent and Crippled Children, Brighton, £67 1s. 8d.; St. Joseph's Convalescent Home, Bournemouth, £62 5s. 10d.; St. Leonards-on-Sea Convalescent Home for Poor Children, £115; St. Mary's Convalescent Home, Shortlands, £17 5s.; St. Michael's Convalescent Home, Westgate-on-Sea, £47 18s. 4d.; Seaside Convalescent Hospital, Seaford, £95 16s. 8d.; Warley Convalescent Cottage Hospital, Essex, £9 11s. 8d.

### 18 COTTAGE HOSPITALS.

Beckenham Cottage Hospital, £67 1s. 8d.; Blackheath and Charlton Cottage Hospital, £69 17s. 5d.; Bromley, Kent, Cottage Hospital, £105 8s. 4d.; Bushey Heath Cottage Hospital, £18 10s. 10d.; Canning Town Cottage Hospital, £47 18s. 4d.; Chislehurst, Sidcup and Cray Valley Cottage Hospital, £51 10s.; Eltham Cottage Hospital, £47 18s. 4d.; Enfield Cottage Hospital, £57 10s.; Epsom and Ewell Cottage Hospital, £62 5s. 10d.; Hounslow Cottage Hospital,

£17 1s. 2d.; Livingstone Cottage Hospital, £86 5s.; Mildmay Cottage Hospital, £38 6s. 8d.; Reigate and Redhill Cottage Hospital, £72 14s. 4d.; Sidcup Cottage Hospital, £38 6s. 8d.; Tilbury (Passmore Edwards) Cottage Hospital, £38 6s. 8d.; Willesden Cottage Hospital, £86 5s.; Wimbledon Cottage Hospital, £52 14s. 2d.; Woolwich and Plumstead Cottage Hospital, £43 2s. 6d.

11 INSTITUTIONS.

Establishment for Gentlewomen, Harley Street, £105 8s. 4d.; S. Saviour's Hospital and Nursing Home, £115; National Sanatorium for Consumption, Bournemouth, £86 5s.; Invalid Asylum, Stoke Newington, £47 18s. 4d.; Firs Home, Bournemouth, £19 3s. 4d.; St. Catherine's Home, Ventnor, £28 15s.; Friedenheim Hospital, Home of Peace, £280 8s.; Oxygen Home, Fitzroy Square, £38 6s. 8d.; St. Francis Hospital, New Kent Road, nil; Santa Claus Home, Highgate, £47 18s. 4d.; St. John's Hospital, Lewisham, £95 16s. 8d.

56 DISPENSARIES.

Battersea Provident Dispensary, £101 11s. 8d.; Blackfriars Provident Dispensary, £18 4s. 2d.; Bloomsbury Provident Dispensary, £14 7s. 6d.; Brixton, etc., Dispensary, £57 10s.; Brompton Provident Dispensary, £21 1s. 8d; Camberwell Provident Dispensary, £83 7s. 6d.; Camden Town Provident Dispensary, £18 4s. 2d.; Chelsea, Brompton, and Belgrave Dispensary, £49 6s. 8d.; Chelsea Provident Dispensary, £14 7s. 6d.; Childs Hill Provident Dispensary, £11 19s. 7d.; City Dispensary, £100 12s. 6d.; City of London and East London Dispensary, £26 16s. 8d.; Clapham General and Provident Dispensary, £28 15s.; Deptford Medical Mission, £23 19s. 2d.; Eastern Dispensary, £47 18s. 4d.; East Dulwich Provident Dispensary, £38 6s. 8d.; Farringdon General Dispensary, £52 14s. 2d.; Finsbury Dispensary, £52 14s. 2d.; Forest Hill Dispensary, £45 0s. 10d.; Greenwich Provident Dispensary, £27 15s. 10d.; Hackney Provident Dispensary, £14 7s. 6d.; Hampstead Provident Dispensary, £47 18s. 4d.; Holloway and North Islington Dispensary, £63 5s.; Islington Dispensary, £59 17s. 11d.; Kensal Town Provident Dispensary, £12 9s. 2d.; Kensington Dispensary, £71 17s. 6d.; Kilburn, Maida Vale and St. John's Wood Dispensary, £45 0s. 10d.; Kilburn Provident Medical Institution, £35 9s. 2d.; Leman Street Provident Dispensary, nil; London Dispensary, Spitalfields, £18 4s. 2d.; London Medical Mission, Endell Street, £119 15s. 10d.; Metropolitan Dispensary, £54 12s. 6d.; Notting Hill Provident Dispensary, £10 10s. 10d.; Paddington Provident Dispensary, £35 9s. 2d.; Portland Town Dispensary, £29 14s. 2d; Public Dispensary, Clare Market, W.C., £49 16s. 8d.; Queen Adelaide's Dispensary, £25 17s. 6d.; Royal General Dispensary, £29 14s. 2d.; Royal Pimlico Provident Dispensary, £59 8s. 4d.; Royal South London Dispensary, £47 18s. 4d.: St. George's and St. James's Dispensary, £57 10s.; St. George's, Hanover Square, Provident Dispensary, £34 10s.; St. John's Wood Provident Dispensary, £42 3s. 4d.; St. Marylebone General Dispensary, £38 6s. 8d.; St. Pancras and Northern Dispensary, £35 9s. 2d.; South Lambeth, Stockwell, and North Brixton Dispensary, £51 15s.; Stamford Hill, Stoke Newington Dispensary, £69; Tower Hamlets Dispensary, £47 18s. 4d.; Walworth Provident Dispensary, £15 6s. 8d.; Wandsworth Common Provident Dispensary, £9 11s. 8d.; Westbourne Provident Dispensary, £16 5s. 10d.; Western Dispensary, £67 1s. 8d.; Western General Dispensary, £143 15s.; Westminster General Dispensary, £42 3s. 4d.; Whitechapel Provident Dispensary, £29 14s. 2d.; Woolwich Provident Dispensary, £14 7s. 6d.

